# Two-Electrode ECG for Ambulatory Monitoring with Minimal Hardware Complexity

**DOI:** 10.3390/s20082386

**Published:** 2020-04-22

**Authors:** Branko Babusiak, Stefan Borik, Maros Smondrk

**Affiliations:** Department of Electromagnetic and Biomedical Engineering, University of Zilina, 01026 Zilina, Slovakia; stefan.borik@feit.uniza.sk (S.B.); maros.smondrk@feit.uniza.sk (M.S.)

**Keywords:** ECG, two-electrodes, hardware design, noise suppression, current consumption, driven right leg

## Abstract

This article introduces a two-electrode ground-free electrocardiogram (ECG) with minimal hardware complexity, which is ideal for wearable battery-powered devices. The main issue of ground-free measurements is the presence of noise. Therefore, noise suppression methods that can be employed for a two-electrode ECG acquisition system are discussed in detail. Experimental measurements of a living subject and patient simulator are used to investigate and compare the performance of the three proposed methods utilizing the ADS1191 analogue front-end for biopotential measurements. The resulting signals recorded for the simulator indicate that all three methods should be suitable for suppressing power-line noise. The Power Spectral Density (PSD) of the signals measured for a subject exhibits differences across methods; the signal power at 50 Hz is −28, −24.8, and −26 dB for the first, second, and third method, respectively. The digital postprocessing of measured signals acquired a high-quality ECG signal comparable to that of three-electrode sensing. The current consumption measurements demonstrate that all proposed two-electrode ECG solutions are appropriate as a battery-powered device (current consumption < 1.5 mA; sampling rate of 500 SPS). The first method, according to the results, is considered the most effective method in the suppression of power-line noise, current consumption, and hardware complexity.

## 1. Introduction

Nowadays, an ageing society is one of the problems emerging on a global scale. The demographics of the global human population from the last decades have revealed that the fraction of individuals older than 60 years increased from 9.2% in 1990 to 11.7% in 2013 and it is expected to reach 21.1% (2 billion) by 2050 [1]. In light of this trend, it can be assumed that conventional healthcare in terms of a hospital-centric concept will not be able to keep up with future demands. Therefore, the concept of preventive healthcare or home healthcare has become a promising solution, which allows us to regularly and systematically measure our health status in an environment outside of hospitals (i.e., at home, in the office, etc.). Monitoring devices should be small, low-power, portable, and in wearable forms [2,3,4]. According to the World Health Organization, cardiovascular diseases are the leading cause of mortality worldwide [5]. The elderly also have high risks of cardiovascular disease, and as a person’s age increases, it becomes more problematic. The symptoms of cardiovascular diseases are uncertain or intermittent. Therefore, it is preferable to monitor a person’s heart activity at any time or place when the symptoms occur. The electrocardiogram (ECG) is the most common diagnostic modality of cardiovascular diseases. The ECG is capable of detecting almost any kind of heart abnormality at an early stage and represents an essential tool for assessment of the cardiovascular system [6]. The twelve-electrode ECGs (e.g., Holter) are considered the gold standard in clinical practice [7]. However, three-electrode ECGs provide a sufficient sensitivity (≈98%) and specificity (≈74%) for distinguishing between native and pathological heart electrical activity [8,9]. In this configuration, two electrodes are used to measure a body surface’s potential difference, and a third electrode provides a low-impedance return path for noise reduction. It is desirable to have few electrodes, in order to reduce the costs of the ECG acquisition system and increase patient comfort. However, the removal of the third electrode is challenging due to the significantly higher electromagnetic interference (EMI) and lower signal-to-noise ratio (SNR) in two-electrode compared to three-electrode ECG acquisition systems [10].

**Figure 1:**
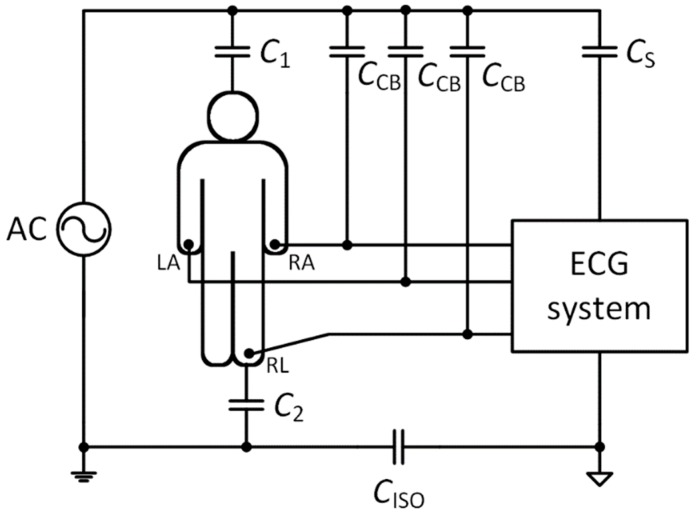
Block diagram of the power-line interference in the three-electrode electrocardiogram (ECG) acquisition system (inspired by [11]).

Biopotential recordings, such as the ECG, are frequently contaminated with 50 Hz (in Europe and most of Asia) or 60 Hz (in the USA and Canada) power-line interference. This interference results from the capacitive coupling of the patient’s body and electrode cables, on the one hand, and the power lines, on the other hand. The sources of power-line interference are summarized in Figure 1.

The C_1_ and C_2_ capacitors act as coupling capacitors between the patient’s body and the power line, and the patient’s body and the ground, respectively. The C_S_ capacitor represents the capacitance between the power line and the ECG acquisition system, whilst C_CB_ capacitors symbolize capacitances between the power line and the electrode cables. Another source of noise is due to the different grounding of the ECG acquisition system and the AC power supply, which results in the coupling capacitance *C*_ISO_ between the AC ground and the ground of the ECG acquisition system [11,12]. In Figure 1, the one-lead ECG acquisition system utilizing three electrodes is depicted. It measures the biopotential between electrodes LA (left arm) and RA (right arm). The third RL (right leg) electrode, sometimes called a reference electrode, is used to minimize power-line interference by means of decreasing the common-mode voltage obtained from the patient’s body.

There are some general techniques for enhancing common-mode rejection (CMR). The C_S_ coupling capacitance can be eliminated by placing the ECG acquisition system (i.e., analogue front-end) into a shielded case and the *C*_CB_ cable coupling capacitances can be eliminated by using shielded electrode cables. The effect of the cable shielding was presented in [13,14]. The CMR system can be enhanced by improving the isolation between the device ground and the patient ground (C_ISO_). Therefore, the battery-powered ECG acquisition systems display very high CMR [11]. The removal of noise caused by the coupling capacitors C_1_ and C_2_ is the most crucial step, because the power-line noise is collected from the patient’s body and transferred as a common-mode voltage (*V*_CM_) to the differential amplifier’s inputs. The common-mode signal is a signal that appears simultaneously and in-phase on both amplifier inputs. The powerline noise coming from the body represents the common-mode voltage. It should be effectively mitigated by using both the differential input amplifier with a high common-mode rejection ratio (CMRR) and a high input impedance. The CMRR represents the capability of an amplifier to reject common-mode signals, and it is defined as the ratio between the amplitude of the common-mode signal and the amplitude of an equivalent differential signal [15,16]. Unfortunately, in both inputs, the common-mode signals are not the same due to mismatches in electrode-skin impedances, cable impedances, input protection circuits (typically including resistors, capacitors, diodes, etc.), and amplifier input impedances [11,17]. This transformation of the common-mode voltage into differential-mode voltage (*V*_DM_) interference must also be considered. 

**Figure 2:**
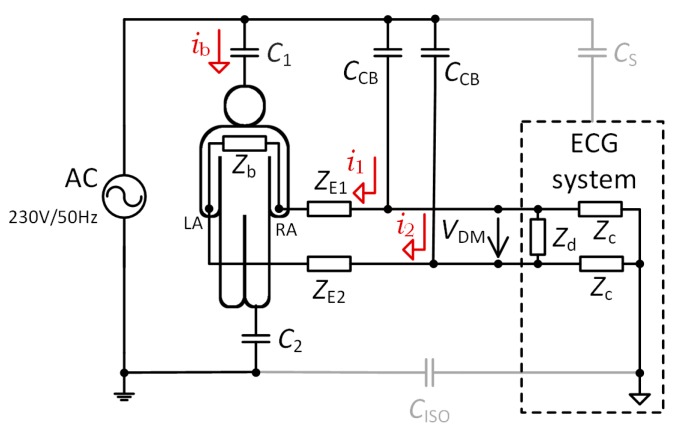
Power-line interference in the two-electrode ECG acquisition system emphasizing differential-mode interference.

Differential-mode (DM) interference is caused by many effects, and it is also the reason why the shielded cables are needed. Unshielded cables suffer from power line interferences through the *C*_CB_ capacities shown in Figure 2. The current flows from the mains power line through the *C*_CB_ capacities, the electrode-skin impedances *Z*_E1_ and *Z*_E2_, and the *C*_2_ body-ground capacitance to the ground, while generating a *V*_DM_ voltage difference between the electrodes due to the different electrode-skin impedances (*Z*_E1_ ≠ *Z*_E2_). This type of interference is known as DM interference [18,19]. 

The differential voltage *V*_DM_ can be calculated as follows [18,20]:

(1) $$V_{\mathrm{DM}}=Z_{E1}i_{1}-Z_{E2}i_{2},$$

where *i*_1_ and *i*_2_ are displacement currents coupled to the electrode leads. The *V*_DM_ voltage is dependent on the distance between power lines and electrode cables and the length of the electrode cables. If the length of the electrode leads is the same, and the leads run close together, then the displacement currents will be equal (*i*_1_ = *i*_2_). If the *i*_1_ and *i*_2_ currents in Figure 2 have a typical value of 10 nA_p-p_ and the imbalance in electrode-skin impedances (*Z*_e1_-*Z*_e2_) is only 20 kΩ, then the magnitude of the bipolar signal in the ECG system input at 50 Hz will be as large as 200 µV_p-p_ [21]. According to the AAMI EC 11 standard, the maximum system noise allowed is 30 µV_p-p_ for an ECG. In practice, it is not possible to have *i*_1_ = *i*_2_ because leads cannot run close together side by side, so *i*_1_ ≠ *i*_2_. If we balance electrode-skin impedances so that *Z*_E1_ = *Z*_E2_, e.g., by the method described in [20], the *V*_DM_ differential voltage will not be zero in Equation (6), because leads have different capacitive couplings of *C*_CB_ and thus, different currents flow to the leads. The cable shielding ensures that power-line currents cannot flow to the electrode leads, and the interference is maximally transformed into a common-mode signal. A previous study [14] showed that the cable shielding increased the attenuation of power-line noise by 19.3 dB, which is why the shielded cables were used in our experiments.

Another source of DM interference is the *i*_b_ displacement current flowing into the body from the power line through *C*_1_ and C*_2_* capacitance to the ground. If we assume typical values of 3 and 300pF for *C*_1_ and C*_2_*, respectively, then an *i*_b_ current of less than 1 µA_p-p_ flows from the power line through the body to the ground [21]. The model situation in Figure 2 assumes that some fraction of the *i*_b_ current is flowing along the *Z*_b_ internal body impedance. This impedance is dependent on the patient orientation and position concerning the power line cables [22]. Then, the DM interferencing voltage caused by *Z*_b_ impedance is

(2) $$V_{\mathrm{DM}}=Z_{b}\cdot i_{b}.$$

If using the maximal values *i*_b_ = 1 µA_p-p_ and *Z*_b_ = 500 Ω, then *V*_DM_ = 0.5 mV_p-p_, which is at a voltage level of the measured ECG signal [19]. We can reduce the DM interference by balancing the electrode and amplifier common-mode input, but there is still some portion of remaining interference because of *Z*_b_. 

A total input interfering voltage *V*_i_ is defined as follows [22,23]:

(3) $$V_{i}=V_{\mathrm{DM}}+V_{\mathrm{CM}}\left(\frac{1}{CMRR_{1}}+\frac{1}{CMRR_{2}}\right),$$

where *V*_DM_ and *V*_CM_ are the differential-mode and common-mode voltages, respectively. Both voltages are the product of the *i*_b_ displacement current. The *CMRR*_1_ in (3) describes the effect of the electrode impedance imbalance Δ*Z*_E_ = *Z*_E1_ – *Z*_E2_:

(4) $$CMRR_{1}=\frac{Z_{C}}{\Delta Z_{E}},$$

where *Z*_c_ is the amplifier CM input impedance at the power line frequency (see Figure 2). A high differential-mode impedance of the amplifier is represented by *Z*_d_. A typical value of *CMRR*_1_ is 60 dB for shielded electrode leads [24]. The *CMRR*_2_ in (3) is a common-mode rejection ratio of a used amplifier (95 dB for ADS1191 used in this paper). The typical value of *V*_CM_ can range from millivolts to tens of millivolts, but it can reach a value of 200 mV_p-p_ [21,22,24]. In general, if we use *V*_DM_ = 0.5 mV_p-p_, *V*_CM_ = 10 mV_p-p_, *CMRR*_1_ = 60 dB, and *CMRR*_2_ = 90 dB, then the total interference voltage *V*_i_ according to (3) will be 510 μV_p-p_. If we change the value of *V*_CM_ to 200 mV_p-p_, then *V*_i_ will be 704 μV_p-p_.

Although a differential amplifier with a high CMRR is used, the differential amplifier does not completely suppress the noise. Moreover, a very high magnitude of the noise can cause saturation of the amplifier input and then it is impossible to extract an ECG from a noisy signal. An additional noise suppression method must then be used. A Driven-Right-Leg (DRL) circuit is often used to reduce the common-mode voltage. The DRL circuit senses the input common-mode voltage at the differential amplifier inputs [25] or outputs [11]. It enhances the CMRR by driving the right leg (RL) electrode through the inverting amplifier, acting as a low-pass filter. In acquisition systems using three electrodes, noise suppression using the DRL circuit is very efficient. In contrast, due to the missing third reference electrode, the power-line noise reduction in the two-electrode system is more complicated. Such a system is usually a portable, battery-powered acquisition system that records the ECG signal from two measuring points located on various body parts, e.g., palms [26], wrists [27,28], and thumbs [29,30,31]. Additionally, these systems usually use contactless gel-free (i.e., dry) electrodes, resulting in increased interference in ECG recordings.

The research community has already published many power-line noise suppression solutions for the two-electrode ECG system [30,31,32]. The research papers present electrical schemes consisting of different operational amplifier combinations and discrete components. However, they could not significantly reduce the overall size of the ECG system, which is a crucial parameter for such a system. Therefore, we focused on using a commercial analogue front-end ECG chip with noise suppression solutions adopted from published research papers. In this paper, we present a comparison of three noise suppression methods for two-electrode ECG system design. The remainder of this paper is organized as follows. Section 2 details our design in terms of the measurement system architecture, hardware, and software required. Section 3 reports the performance of the noise suppression methods, experiments with a simulator and living subjects, measurement outcomes, and current consumption of the proposed solutions. Finally, conclusions are drawn in Section 4.

## 2. Materials and Methods

We decided to use an ADS1191 analogue front-end designed for the two-electrode measurement of ECG. The ADS1191 is a low-power integrated analogue front-end for ECG application from Texas Instruments. This version of ADS contains one input channel of a 16-bit delta-sigma analogue-to-digital converter with a built-in programmable gain amplifier, internal reference, and on-board oscillator. It operates at data rates up to 8 kSPS. It includes a built-in Driven-Right-Leg amplifier, lead-off detection, and a test signal generator. ADS1191 enables the creation of one-channel ECG systems with a significantly reduced size (5 × 5 mm2), power, and overall cost. The most critical parameters for the design of the two-electrode ECG system are the CMRR of 95 dB and the presence of a DRL circuit [33].

**Figure 3:**
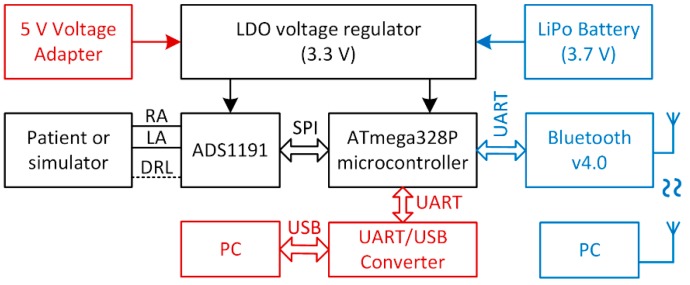
Block diagram of the measurement system. A red scenario stands for powering the ECG system by an AC/DC adapter where data are transferred to the PC via a USB cable. A blue scenario stands for a battery-powered ECG system where data are transferred wirelessly via Bluetooth.

The ADS1191 (ADS) is controlled by the ATmega328P microcontroller (MCU) via the Serial Peripheral Interface (SPI). The parameters, such as the sampling rate, gain of the differential amplifier, and utilization of the DRL circuit, can be set by the MCU. The ADS and MCU are powered by a low-dropout (LDO) voltage regulator connected to a lithium polymer (LiPo) battery. Consequently, the sampled data collected by the MCU are sent to a PC via a USB interface using a UART to USB converter. The measured data are then analyzed by the PC using MATLAB (ver. 9.5.0, MathWorks, USA). A block diagram of the measurement system is shown in Figure 3. The vital signs monitor simulator ProSim 2 (FLUKE, USA) was used for the generation of the bipolar input signal. The ProSim 2 simulator can generate ECG signals with a defined QRS complex magnitude and desired heart rate. It can also generate artificial signals such as a square, triangle, sequence of pulses, and sine wave with a desired amplitude and frequency. The sine wave signal with a frequency of 5 Hz and amplitude of 1 mV was brought to the ADS1191 inputs from the simulator. The simulator was powered by an independent battery. The signal was sampled at 500 SPS. If we use a sine wave signal in the input, then the calculation of the SNR of the ADS1191 output is straightforward.

**Figure 4:**
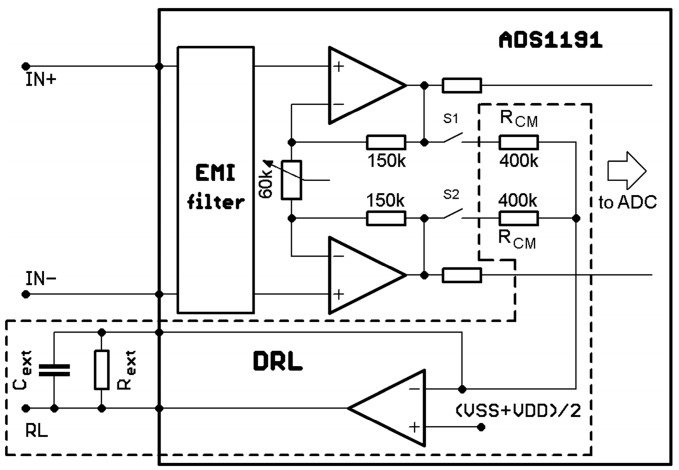
Part of a functional block diagram with the Driven-Right-Leg (DRL) circuit.

A functional block diagram of the part of interest is shown in Figure 4. The part of interest shows the electrical circuit, which is responsible for power-line noise suppression—the DRL circuit. This circuit senses the common-mode signal from the instrumentation amplifier (integrated within ADS1191) by 400 kΩ resistors (R_CM_) and then drives the right leg (RL) through the inverting amplifier. The DRL circuit can be programmatically connected or disconnected by the S1 and S2 switches.

The gain and cut-off frequency of the feedback DRL loop are adjusted by an external R_ext_ resistor and C_ext_ capacitor. The ***G*** gain of the DRL amplifier is computed as [11]:

(5) $$G=-2\cdot \frac{Z_{F}}{R_{\mathrm{CM}}},$$

where ***Z***_F_ is frequency-dependent, and it is a parallel combination of the R_ext_ resistor and the C_ext_ capacitor:

(6) $$Z_{F}=\frac{R_{\mathrm{ext}}}{1+j\omega R_{\mathrm{ext}}C_{\mathrm{ext}}}.$$

Combining Equations (5) and (6) leads to the following equation, which describes the overall gain of the DRL amplifier:

(7) $$G=-2\cdot \frac{\frac{R_{\mathrm{ext}}}{1+j\omega R_{\mathrm{ext}}C_{\mathrm{ext}}}}{R_{\mathrm{CM}}}=\left(-2\cdot \frac{R_{\mathrm{ext}}}{R_{\mathrm{CM}}}\right)\cdot \left(\frac{1}{1+j\omega R_{\mathrm{ext}}C_{\mathrm{ext}}}\right)=A\cdot \left(\frac{1}{1+j\omega R_{\mathrm{ext}}C_{\mathrm{ext}}}\right),$$

where *A* represents the overall gain of the DRL amplifier in the passband region. The cut-off frequency of the low-pass filter of the DRL loop is defined as

(8) $$f_{c}=\frac{1}{2\pi R_{\mathrm{ext}}C_{\mathrm{ext}}}.$$

### 2.1. Three-Electrode ECG Acquisition System

**Figure 5:**
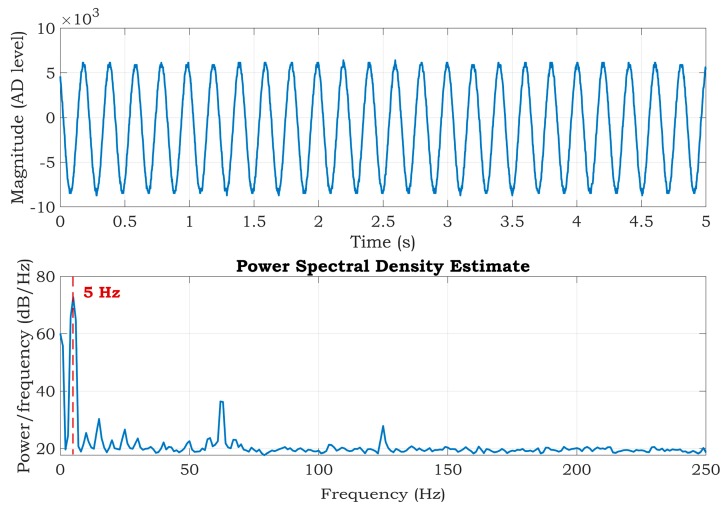
The first 5 s of the output signal (top) and Power Spectral Density (PSD) estimate (bottom) of the three-electrode system with an enabled DRL circuit.

To obtain a reference, a three-electrode ECG system was firstly used to compare the three-electrode with the two-electrode ECG system. The IN+ and IN- inputs of ADS1191 were connected to the simulator LA and RA outputs, and the third electrode—the output of the DRL circuit (RL)—was connected to the RL output of the simulator. The *R*_ext_ and *C*_ext_ values were 1 MΩ and 1.5 nF, respectively. The *f*_c_ cut-off frequency was then about 106 Hz, and the *G* gain of the DRL amplifier calculated by Equation 7 was approximately −4.5 for a frequency of 50 Hz. The values of *R*_ext_ and *C*_ext_ were adopted from the typical application circuit of the ADS1191. Although the gain value is small in comparison with some other studies, e.g., [34] uses an approximately ten times higher DRL gain, the analogue front-ends with relatively high common-mode rejection need low common-mode reduction by the DRL circuit [24]. This fact is also supported by measurements provided in the results section. The measured signal and its Power Spectral Density (PSD) estimate are shown in Figure 5. The PSD estimation was based on the Welch method. The Hamming window with a length of one second and 50% window overlap was used for PSD estimation. The signal was measured for 1 min, but only the first 5 s are shown in Figure 5. It can be seen that there is no noise at the power-line frequency (50 Hz), but there is still some background noise from the environment.

The SNR is often used to compare the level of the desired signal to the level of background noise. We calculated the SNR from one-sided PSD according to the following equation:

(9) $$\mathrm{SNR}=10{\;\log}_{10}\frac{P_{\mathrm{signal}}}{P_{\mathrm{noise}}}=P_{\mathrm{signal},\mathrm{dB}}-P_{\mathrm{noise},\;\mathrm{dB}}\;\left[dB\right],$$

where *P*_signal_ is the power of the carrier sinusoidal input signal (5 Hz), and *P*_noise_ is the power of background noise, excluding the DC component and the first two harmonics (multiples of the carrier signal). The calculated SNR of the signal was equal to 28.73 dB (Figure 5).

**Figure 6:**
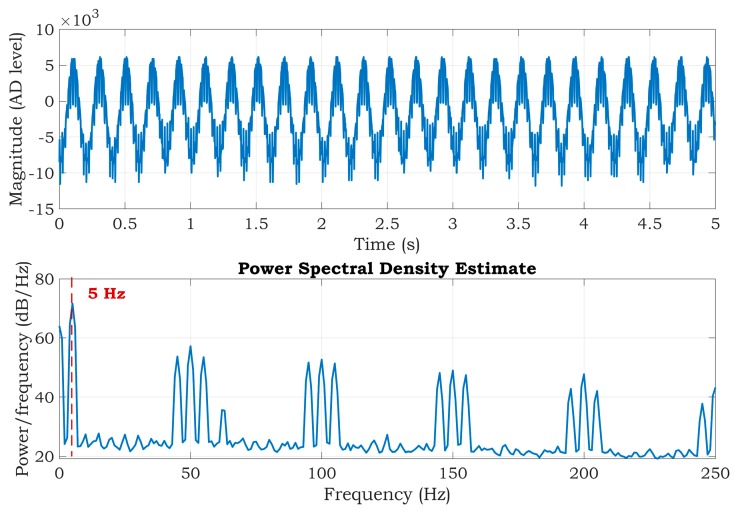
The first 5 s of the output signal (top) and PSD estimate (bottom) of the three-electrode system with a disabled DRL circuit.

The situation without a DRL circuit is depicted in Figure 6. The DRL circuit was disabled by disconnecting the RL electrode from the simulator. The power-line noise is presented at a frequency of 50 Hz and its frequency multiples. Moreover, further noise was added to the signal at a power-line frequency ±5 Hz (see Figure 6). The same stands for the multiples of the power-line frequency. The calculated SNR was 8.83 dB. A comparison of Figure 5 and Figure 6 shows the effectivity and importance of the DRL circuit in the ECG measurement.

### 2.2. Two-Electrode ECG Acquisition System

There are many designs of a two-electrode system for ECG measurement [26,27,28,29,30,31,32]. We selected two types of solutions for mains noise suppression in a two-electrode ECG system from the published research papers and applied them for the analogue ECG front-end embedded in one chip. The selected solutions are characterized by a reduced hardware complexity, which is desirable for small portable acquisition devices.

**Figure 7:**
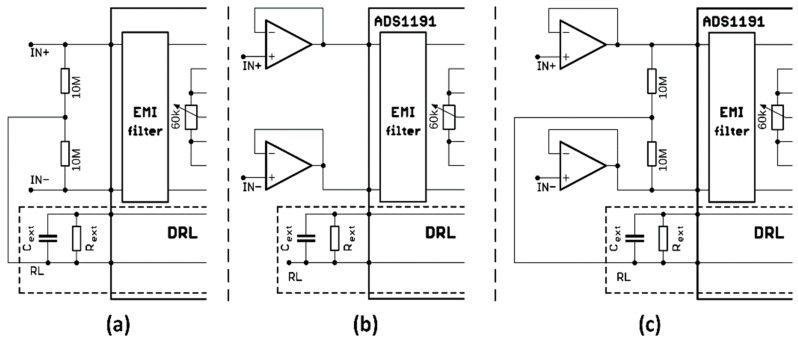
Proposed solutions for a two-electrode ECG system (**a**) using DRL with 10 MΩ resistors, (**b**) using active electrodes, and (**c**) using a combination of previous solutions.

The first of the solutions was presented in [23,31,32]. The output from the DRL circuit is connected to input electrodes through resistors with a large value of resistance. This solution is presented in Figure 7a. Therefore, we biased the inputs IN+ and IN- by the output of the DRL circuit through 10 MΩ resistors. The value of these resistors should be high (≈MΩ) because they limit the current going to the patient through the DRL circuit. The inputs IN+ and IN- were connected to the simulator outputs RA and LA through one-meter-long shielded cables with shielding connected to the signal ground.

Another unique solution of the two-electrode ECG system was presented in [30]. This solution does not use DRL for measurement of the ECG from the thumbs by using gel-less, dry electrodes. It uses a unity-gain amplifier (buffer) integrated on the back-side of the electrode face. The buffer converts the high impedance of the dry electrode to low output impedance, which helps to isolate the electrodes from ADS1191 inputs and reduce interference from the background. Moreover, voltage followers on the inputs compensate for differences in electrode-skin impedance between two measured points on the body. This feature helps to substantially increase the CMRR. A similar solution is used in capacitive sensing of the ECG signal [35]. In the case of capacitive signal sensing, the backside of the electrode is shielded due to noise protection. This type of electrode is called the active electrode. The signal from active electrodes continues to the inputs of the instrumentation amplifier (Figure 7b).

**Figure 8:**
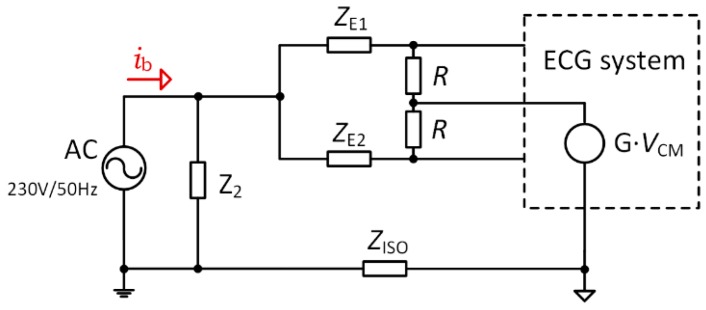
Equivalent circuit of the interference model using DRL.

In the case of using the DRL circuit for common-mode suppression (Figure 7a), the interference model depicted in Figure 2 will be transformed into model in Figure 8. This model was introduced by the authors in [23], from which our solution was adopted. An *i*_b_ displacement current is branched into currents flowing to the body through the *Z*_2_ impedance and to the ECG system through the *Z*_E1_ and *Z*_E2_ electrode impedances. The *Z*_2_ and the *Z*_ISO_ impedances represent reactances of coupling capacitances of *C*_2_ and *C*_ISO_, respectively. 

In [23], it was shown that the *Z*_c_ common-mode impedance is controlled by common-mode feedback gain *G* of the DRL circuit:

(10) $$Z_{c}=\frac{R}{1-G}\;.$$

Using a high value of *R* ensures a high differential-mode input impedance of *Z*_d_, which is equal to 2*R*. We used *R* = 10 MΩ and a gain of DRL of *G* = −4.5. Therefore, *Z*_c_ = 1.8 MΩ, according to Equation (10). The total input interfering voltage *V*_i_ for the model shown in Figure 8 is [23]

(11) $$V_{i}=i_{b}\frac{Z_{2}}{Z_{c}+2\left(Z_{2}+Z_{\mathrm{ISO}}\right)}\left(\Delta Z_{E}+\frac{Z_{c}}{CMRR_{2}}\right).$$

According to the experimental measurements and graph provided in [23], in which it is assumed that *i*_b_ is approx. 105 nA, *CMRR*_2_ = 90 dB (similar to the CMRR of ADS1191), and our *Z*_c_ = 1.8 MΩ, the total input interference *V*_i_ will be about 52.5, 78.8, and 131.3 μV for Δ*Z*_E_ = 10, 20, and 40 kΩ, respectively.

In [30], the INA333 (Texas Instruments, USA) is used as a differential amplifier with a relatively high CMRR of 100 dB. The ADS1191 has a very similar CMRR (95 dB). The DRL circuit of ADS1191 was programmatically disabled by disconnecting the S1 and S2 switches, as shown in Figure 4. We used OPA2333 (Texas Instruments, USA) as an input buffer. The OPA2333 integrates two operational amplifiers of OPA333 into one package. The simulator was connected to the inputs in the same manner as in the previous case, by one-meter-long shielded cables.

The third tested solution is a combination of voltage followers on the inputs and output of the DRL circuit connected to the inputs via 10 MΩ resistors (Figure 7c). This solution is similar to a solution used in [36]. The DRL circuit of ADS1191 was programmatically enabled, and the simulator was connected to the inputs by one-meter-long shielded cables. However, whilst the shielded cable is not necessary for the first solution, it is crucial for the second and third solution. In contrast to the first situation (Figure 7a), we were not able to measure the ECG signal without shielding.

## 3. Results

**Figure 9:**
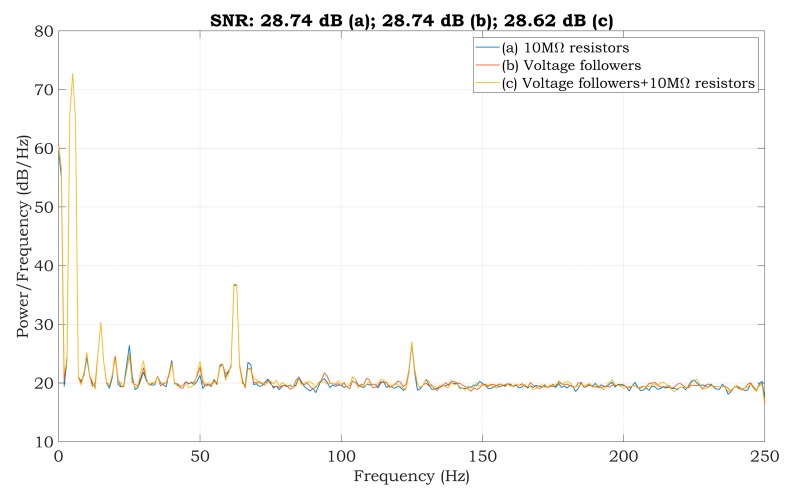
The PSD estimate of the sinewave signal of proposed solutions.

We tested the practical implementation of the proposed solutions for the two-electrode ECG system. The one-minute-long sine wave signal with a frequency of 5 Hz and amplitude of 1 mV was measured while being generated by the FLUKE ProSim 2 simulator (FLUKE Biomedical, Cleveland, USA). The PSD estimates of measured signals are shown in Figure 9. 

The PSD estimates are very similar for all three cases with very close values of the SNR. The calculated SNRs are shown in the title of Figure 9. The frequency component of 50 Hz in the power spectrum is reduced when compared to the spectrum in Figure 6. Furthermore, the power spectrum is very similar to the spectrum of the system, which uses three electrodes (Figure 5). According to the results, we can suggest that all three alternatives should be suitable for suppressing power-line noise in two-electrode ECG systems.

**Figure 10:**
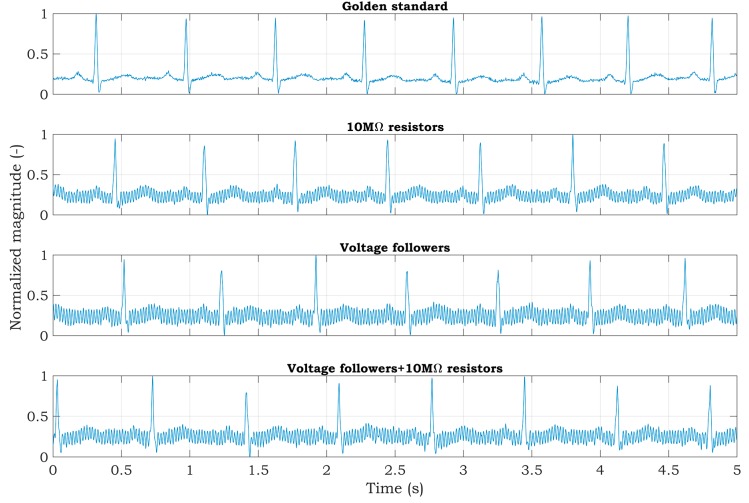
Raw ECG signals measured from a living subject.

**Figure 11:**
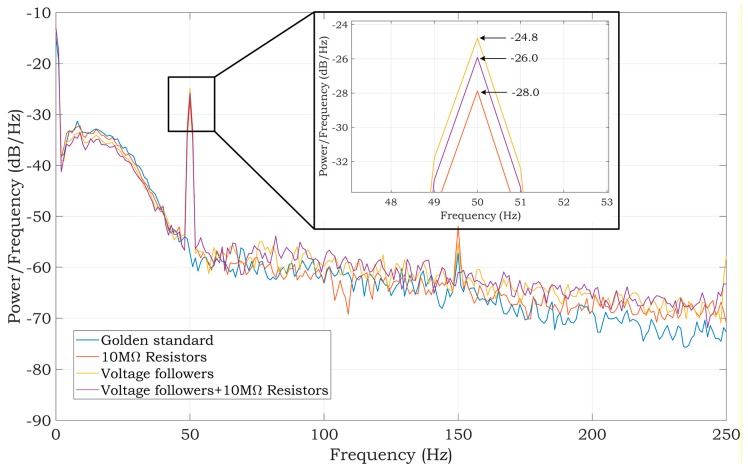
PSD estimates of raw ECG signals measured from a living subject. The power of the golden standard signal at 50 Hz is −56.67 dB.

Experiments with a living subject were also performed, where ECG clamp electrodes were placed on the proband wrists. The methods, according to Figure 7, for active noise suppression, were used to avoid saturation of the inputs. The ECG system was placed in the shielded box and powered by a 5 V voltage adapter (a red scenario in Figure 3). The ECG signal measured from three electrodes by the BIOPAC MP36 acquisition system (BIOPAC Systems, USA) is considered to be the golden standard. The DRL electrode was placed on the ankle of the right leg. All measurements were provided for the same subject in sequence, with the minimum idle time between them. The sampling rate was set to 500 SPS for all measurements. Measured signals are shown in Figure 10. The magnitude of signals was normalized to the interval from 0 to 1 to compare the shape of the ECG signal and the amount of noise between specific hardware solutions. At first sight, minimal noise is visible in the solution with 10 MΩ resistors on the ADS inputs. This fact is also confirmed by the PSD estimation shown in Figure 11. Although only five seconds of signals are shown in Figure 10, the PSD in Figure 11 was calculated for one-minute-long signals. The PSD shows the presence of the power-line noise in all cases, except for the golden standard, which was not high enough to cause the differential amplifier inputs to be saturated. The power of the signal at a frequency of 50 Hz was −28.0, −24.8, and −26.0 dB for the solution with 10 MΩ resistors, voltage followers, and the combination, respectively. 

Time-domain or frequency-domain analysis, or their combination, may be used to evaluate the signal quality of the acquired ECG [37]. In our case, we used frequency analysis of the signal quality obtained by the gold standard and three proposed solutions of the two-electrode ECG system. We started the work in accordance with [38], dividing the frequency band similarly into three areas:

- Low-Frequency band (LF): 0–0.5 Hz;
- High-Frequency band (HF): 40–250 Hz;
- ECG band: 0.5–40 Hz.

**Figure 12:**
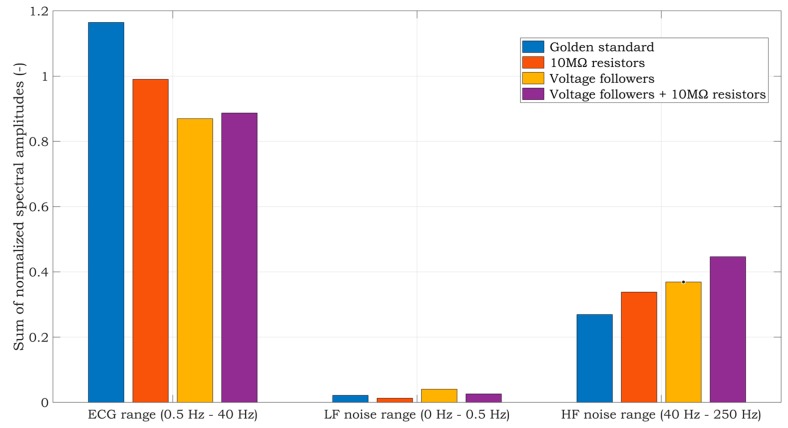
Frequency-domain analysis of raw ECG signals measured from a living subject.

At the same time, the LF band includes isoline changes and artifacts, such as the breath waveform. HF noise contains power-line noise and its higher harmonic components or muscle EMG artifacts. For the analysis, we selected the signal segments with a length of 15 s. Firstly, the DC component was removed from the signals, and then, we normalized them using the maximum value of the R wave. For each signal, the fast Fourier transform (FFT) was calculated using a Hamming window. Subsequently, we summed the components for each frequency band. The result of the analysis is depicted in Figure 12.

Looking at the ECG band, the best result was achieved by the BIOPAC system, which also serves as the gold standard. The worst result was the voltage follower solution. In terms of low-frequency noise contamination, the 10 MΩ resistor system, in combination with a virtual DRL circuit, shows the most considerable LF noise resistance. The high-frequency noise mostly occurs in the fourth solution and thus in the combination of voltage followers and 10 MΩ resistors. This correlates with the PSD shown in Figure 11. As a result, the solution using virtual DRL and 10 MΩ resistors seems to be the best in terms of LF and HF noise, as well as the useful ECG signal. This noise can be additionally removed by several techniques, e.g., implementing a notch filter, wavelet filtering, or changing the DRL circuit’s gain, etc. 

**Figure 13:**
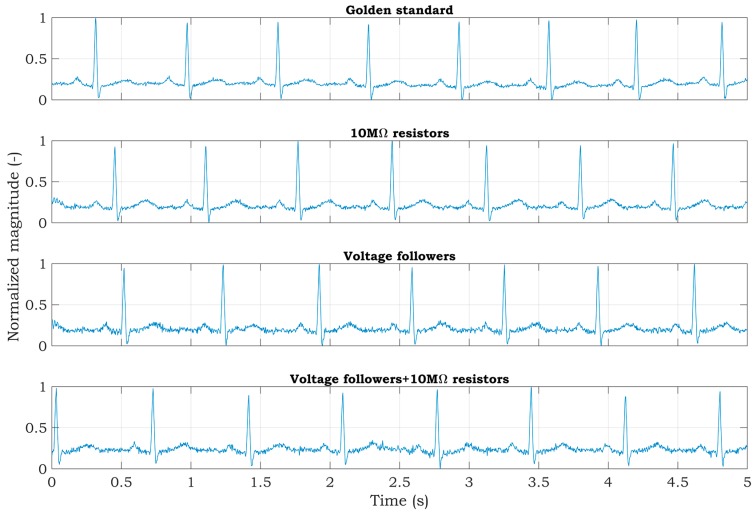
Application of a digital notch filter to ECG signals measured from a living subject.

The raw signals were post-processed to enhance the signal quality and to demonstrate the effectivity of the proposed hardware solutions for high-quality ECG measurement. The signal denoted as the golden standard did not undergo postprocessing in the following figures because the power-line noise was minimal. At first, a notch filter was applied to raw signals. The filter was designed with attenuation of 60 dB at 50 Hz. The resulting signals are shown in Figure 13.

**Figure 14:**
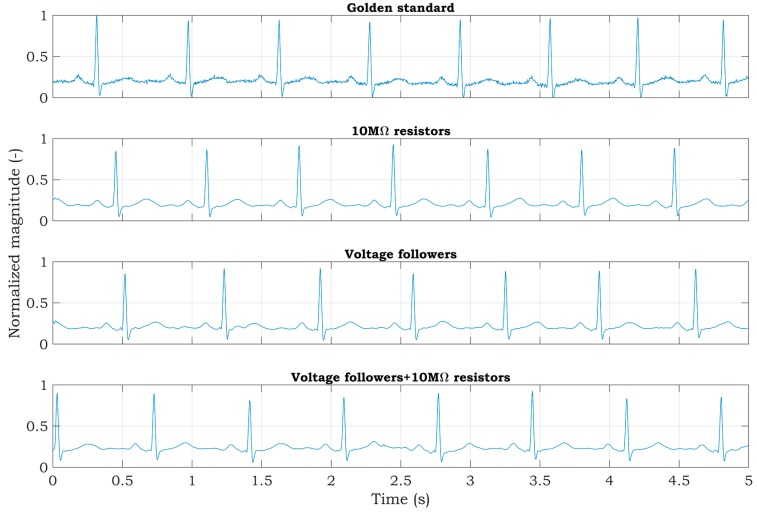
Wavelet filtration of ECG signals measured from a living subject.

After using a notch filter, the signals were further enhanced and smoothed by wavelet denoising. Signals were decomposed into five levels using the *Symlets 4* wavelet and filtered in the wavelet space using soft thresholding. Filtered signals were then composed by using the inverse discrete wavelet transform (Figure 14).

**Figure 15:**
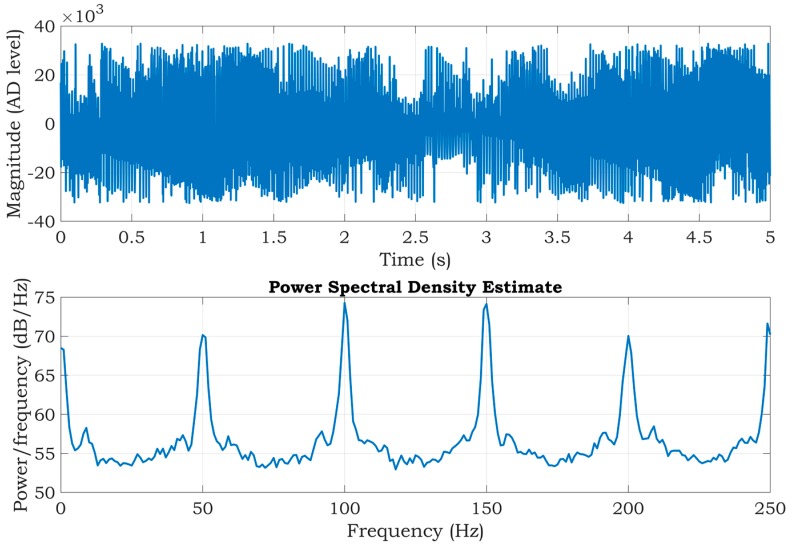
ECG signal measured from a living subject without noise suppression (top), and the corresponding PSD estimate (bottom).

Finally, an experiment was carried out without using any hardware techniques of noise suppression. The resulting signal and its PSD are shown in Figure 15. In this figure, the ECG signal is lost in a high quantity of noise. In some intervals, the noise saturates the inputs of the differential amplifier (signal magnitudes overreached the input range of the differential 16-bit AD converter). Therefore, at the signal postprocessing stage, it is not feasible to fully extract an ECG from such a noisy signal.

## 4. Discussion

Comparing all three hardware solutions for noise suppression in ECG measurement, it is evident that the first solution actively suppresses the common noise component in the form of power-line noise. It also features active compensation for a possible common-mode noise reduction in the form of a DRL circuit when the measuring electrodes have distinct characteristics (i.e., electrical impedance at the interfaces: electrode, gel, and skin). This compensation is also included in the second and third noise suppression solutions, where the voltage followers ensure impedance separation of the measuring circuit and the electrode-gel-skin interfaces.

The cable shielding is necessary for designs where voltage buffers are used on the ECG device inputs (second and third method). Unshielded cables suffer from common-mode and differential-mode interferences through *C*_CB_ capacities (Figure 1 and Figure 2). A high magnitude of such interference can cause saturation of the amplifier inputs and it is then not possible to measure the ECG signal. The cable shielding is not needed in the first method because the DRL circuit provides active noise reduction by driving the inverted and amplified common-mode signal back to the electrode leads. 

Depending on the current physiological state of the subject being measured, the impedance of the electrode-gel-skin interfaces may considerably vary. This fact is also supported by the final measurement (Figure 15), wherein the measured signal was directly applied to the ADS1191 inputs, without any active suppression technique of the common-mode interference or impedance isolation by the voltage followers. The imbalance between both inputs significantly degrades the CMRR and makes it impossible to analyze the measured ECG (even after digital signal filtering) due to saturation of the ADS1191 inputs.

The last part, which must be discussed, is aimed at the current consumption of proposed hardware solutions which are intended to be used as battery-powered devices. The current consumption is measured in the configuration, according to the diagram shown in Figure 3. To reduce the power consumption, the MCU ATmega328P runs at a frequency of 2 MHz, which is sufficient for the highest sampling rate of ADS1191 (500 SPS). The MCU peripherals, such as the AD converter and timers, are powered down. The MCU and ADS1191 are powered by a rechargeable lithium-polymer (LiPo) battery with a nominal voltage of 3.7 V with a capacity of 1200 mAh. The external dimensions of the battery are 10 × 30 × 40 mm, and the weight is only 18 g. These parameters make the battery ideal for wearable devices. The UART/USB converter is powered by an external power source because the converter is not essential in applications when the data are transmitted wirelessly to the receiver, such as a PC, tablet, smartphone, etc. The current consumption was measured by using the digital multimeter Agilent 34401A (Agilent Technologies, USA) placed between the battery and the LDO regulator. The battery had a voltage of 3.68 V during the measurement. Three power modes were investigated, and the current consumption values are shown in Table 1.

The current consumption is slightly higher in the solution with voltage followers because op-amps (OPA2333) used as voltage followers consume a little extra power. The third solution, which is a combination of 10 MΩ resistors and voltage followers, is not mentioned in the table because the consumption does not differ from that of the voltage followers solution. Some current is drawn from the battery when the MCU and ADS1191 are powered down (third variant). This current results in internal consumption of the LDO regulator, external pull-up or pull-down resistors, and quiescent power-down currents of MCU and ADS. The second power mode variant allows us to discover the current consumption of a particular solution, excluding MCU consumption. The current consumption of the solution with 10 MΩ resistors and voltage followers is 187 and 205 µA, respectively. According to the ADS1191 datasheet [33], the power consumption in normal mode with a DRL circuit disabled at 3 V is typically 420 µW, which means a current consumption of 140 µA. The DRL is enabled in the first solution, and additional voltage followers are used in the second solution, so the current consumption is higher and comparable to datasheet values. The current consumption in the first power mode variant is approximately 1.5 mA in both solutions. These values are measured while sending ECG data at 500 SPS over USART to a PC. In most cases, the data from the battery-powered device are transferred wirelessly. The Bluetooth Low Energy (BLE) is aimed at novel applications in healthcare and offers considerably reduced power consumption. As was stated by the authors in [39], the RN4020 BLE module by Microchip drains only 7.6 mA at 3 V while sending data at 500 SPS. This BLE module, in combination with our two-electrode ECG solutions, ensures that the current consumption does not increase above 10 mA. If we consider a battery with a capacity of 1200 mAh, then the proposed ECG device is able to send the data continuously over Bluetooth for 120 h (5 days).

**Figure 16:**
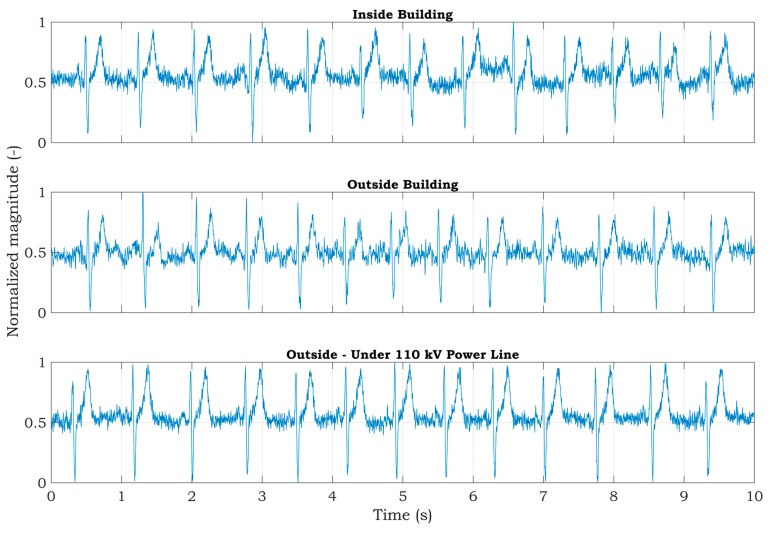
Raw ECG signals measured from a living subject by a battery-powered ECG device using the first power-line noise suppression method.

**Figure 17:**
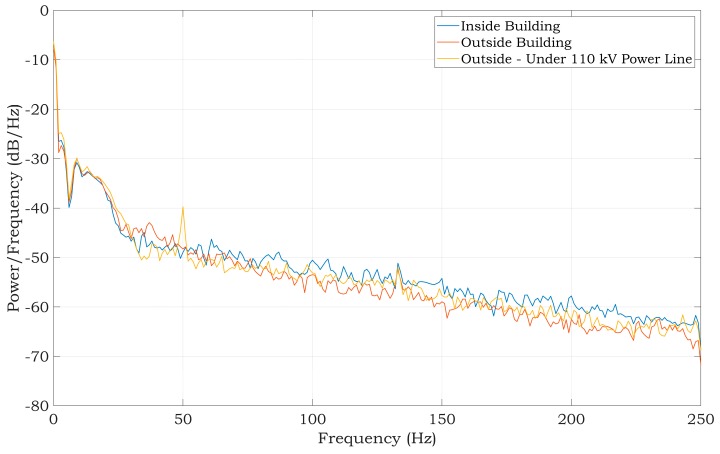
PSD estimates of raw ECG signals measured from a living subject by a battery-powered ECG device using the first power-line noise suppression method.

The best power-line noise suppression was achieved by using the first method with 10 MΩ resistors on the ADS1191 inputs (see Figure 11). The battery-powered ECG system, according to the blue scenario of the measurement system in Figure 3, implementing the first method (Figure 7, case (a)), was tested in three different environments contaminated by power-line noise. The ECG was measured three times for three days and the representative raw signals are shown in Figure 16. The first signal was measured in an office, inside of a university building; the second one was measured outside of the university in a park area; and the last measurement was performed in the exterior, under high-voltage transmission lines (see Figure 17 for ECG waveforms). The PSD estimates of signals are depicted in Figure 16. As can be seen in Figure 17, the 50 Hz power-line frequency is significantly reduced inside and outside of the building, while under the high-voltage cables, it has a power of about 10 dB. The reason for such attenuation of the power-line frequency when compared to PSD in Figure 11 rests in powering the ECG system with a battery, causing high isolation impedance *Z*_ISO_ between the power-line ground and the acquisition system ground (see Figure 8).

## 5. Conclusions

This article aimed to introduce the design of a two-electrode ECG device with minimal hardware complexity. Since the overall size of the ECG acquisition system is a crucial parameter (especially in wearable technologies), the analogue front-end is now a promising solution for overcoming the size limitation of the ECG systems built from discrete components, and differential and operational amplifiers. Therefore, the measuring apparatus includes an analogue front-end controlled by a microcontroller used for sensing the test signal generated by a vital sign simulator and the ECG signal taken from a living subject. The main problem facing the two-electrode system is the noise which is usually eliminated by the third electrode. This paper has introduced noise suppression methods for a two-electrode ECG acquisition system and compared three methods adopted from published research papers. The first method includes the DRL circuit connected by the resistors to the input electrodes. Instead of this circuit, the second method uses unity-gain input amplifiers and the third method implements a combination of previous methods.

The experiments conducted with a patient simulator indicate that all methods should be appropriate for suppressing the power-line noise in two-electrode ECG systems. The estimates of the PSD, while the test sine signal was being measured, achieved very similar SNR values; the SNR for the first and second methods was 28.74 dB and it was 28.62 dB for the third method (Figure 9). According to the measurements obtained with the simulator, it leads to comparable results for the ECG signals measured from a living subject utilizing the mentioned methods (Figure 11). The PSD shows the power of −28, −24.8, and −26 dB at 50 Hz for the first, second, and third method, respectively. The first method’s reduction of power-line noise is about 3 dB and 2 dB better compared to the second and third solution, respectively.

Power consumption is crucial for battery-powered devices. The current consumption is slightly higher for methods using additional op-amps (Table 1), but it does not surpass 1.5 mA. If an appropriate wireless module is used for data transfer, then the current consumption does not surpass 10 mA.

However, due to the two resistors involved and the DRL circuit integrated in ADS1191, the first method features a reduced hardware complexity (lower costs and dimensions) compared to the remaining methods. Moreover, the second and third methods need shielded electrode cables for proper measurements. The experiments provided in this paper show that all of the proposed methods are suitable for high-quality two-electrode ECG measurement (Figure 13). However, the first hardware solution is the most effective because it has the highest mains noise suppression, lowest current consumption, lowest hardware complexity, and no necessity for shielded cables.

## Figures and Tables

**Table 1:** Average current consumption. Battery voltage: 3.68 V.

Power Mode Variants		Current Consumption (mA)	
		10 MΩ Resistors	Voltage Followers
1	MCU (Active) & ADS (Active)	1.425	1.491
2	MCU (Power Down) & ADS (Active)	0.342	0.387
3	MCU (Power Down) & ADS (Power Down)	0.155	0.182

## References

[1] Sander M., Oxlund B., Jespersen A., Krasnik A., Mortensen E.L., Westendorp R.G.J., Rasmussen L.J. (2015). The challenges of human population ageing. Age Ageing.

[2] Axisa F., Schmitt P.M., Gehin C., Delhomme G., McAdams E., Dittmar A. (2005). Flexible technologies and smart clothing for citizen medicine, home healthcare, and disease prevention. IEEE Trans. Inf. Technol. Biomed..

[3] Pantelopoulos A., Bourbakis N.G. (2010). A Survey on Wearable Sensor-Based Systems for Health Monitoring and Prognosis. IEEE Trans. Syst. Man Cybern. Part C (Appl. Rev.).

[4] Appelboom G., Camacho E., Abraham M.E., Bruce S.S., Dumont E.L.P., Zacharia B.E., D’Amico R., Slomian J., Reginster J.Y., Bruyère O. (2014). Smart wearable body sensors for patient self-assessment and monitoring. Arch. Public Heal..

[5] Kishore S.P., Blank E., Heller D.J., Patel A., Peters A., Price M., Vidula M., Fuster V., Onuma O., Huffman M.D. (2018). Modernizing the World Health Organization List of Essential Medicines for Preventing and Controlling Cardiovascular Diseases. J. Am. Coll. Cardiol..

[6] Javorka M., Krohova J., Czippelova B., Turianikova Z., Lazarova Z., Wiszt R., Faes L. (2018). Towards understanding the complexity of cardiovascular oscillations: Insights from information theory. Comput. Biol. Med..

[7] Serhani M.A., T El Kassabi H., Ismail H., Nujum Navaz A. (2020). ECG Monitoring Systems: Review, Architecture, Processes, and Key Challenges. Sensors.

[8] Koltowski L., Balsam P., Glowczynska R., Peller M., Maksym J., Blicharz L., Niedziela M., Maciejewski K., Opolski G., Grabowski M. (2017). Comparison of Kardia Mobile (one lead ECGs records) with 12-lead ECGs in 100 consecutive patients with various cardiovascular disorders. EP Eur..

[9] Ye-Lin Y., Bueno-Barrachina J.M., Prats-boluda G., Rodriguez de Sanabria R., Garcia-Casado J. (2017). Wireless sensor node for non-invasive high precision electrocardiographic signal acquisition based on a multi-ring electrode. Meas. J. Int. Meas. Confed..

[10] Wood D.E., Ewins D.J., Balachandran W. (1995). Comparative analysis of power-line interference between two- or three-electrode biopotential amplifiers. Med. Biol. Eng. Comput..

[11] Acharya V. (2011). Improving Common-Mode Rejection Using the Right-Leg Drive Amplifier.

[12] Lim Y.G., Chung G.S., Park K.S. Capacitive driven-right-leg grounding in indirect-contact ECG measurement. Proceedings of the 2010 Annual International Conference of the IEEE Engineering in Medicine and Biology Society, EMBC’10.

[13] Jiang Y., Ji N., Wang H., Liu X., Geng Y., Li P., Chen S., Li G. Comparison of different shielding methods in acquisition of physiological signals. Proceedings of the Annual International Conference of the IEEE Engineering in Medicine and Biology Society, EMBS.

[14] Jiang Y., Samuel O.W., Liu X., Wang X., Idowu P.O., Li P., Chen F., Zhu M., Geng Y., Wu F. (2018). Effective biopotential signal acquisition: Comparison of different shielded drive technologies. Appl. Sci..

[15] Prutchi D., Norris M. (2005). Design and Development of Medical Electronic Instrumentation: A Practical Perspective of the Design, Construction, and Test of Medical Devices.

[16] Serrano-Finetti E., Casas O., Pallàs Areny R. (2019). Common mode electronic noise in differential circuits. Meas. J. Int. Meas. Confed..

[17] Vozda M., Hrvolova B., Krohova J., Smondrk M., Penhaker M. (2013). Computer-Based Vectorcardiograph for Research Purposes. Electron. Electr. Eng..

[18] Becchetti C., Neri A. (2013). Medical Instrument Design and Development: From Requirements to Market Placements.

[19] Díaz D., Casas Ó., Pallàs-Areny R. (2009). Interference reduction in ECG recordings by using a dual ground electrode. Proceedings of the 19th IMEKO World Congr 2009.

[20] Yamamoto Y. Impedance balancing analysis for power-line interference elimination in ECG signal. Proceedings of the Conference Record—IEEE Instrumentation and Measurement Technology Conference.

[21] Metting van Rijn A.C., Peper A., Grimbergen C.A. (1990). High-quality recording of bioelectric events—Part 1 Interference reduction, theory and practice. Med. Biol. Eng. Comput..

[22] Chimeno M.F., Pallàs-Areny R. (2000). A comprehensive model for power line interference in biopotential measurements. IEEE Trans. Instrum. Meas..

[23] Spinelli E.M., Mayosky M.A. (2005). Two-electrode biopotential measurements: Power line interference analysis. IEEE Trans. Biomed. Eng..

[24] Haberman M.A., Spinelli E.M. (2012). A multichannel EEG acquisition scheme based on single ended amplifiers and digital DRL. IEEE Trans. Biomed. Circuits Syst..

[25] Winter B.B., Winter B.B. (1983). Driven-Right-Leg Circuit Design. IEEE Trans. Biomed. Eng..

[26] Richard E., Chan A.D.C. Design of a gel-less two-electrode ECG monitor. Proceedings of the 2010 IEEE International Workshop on Medical Measurements and Applications, MeMeA 2010—Proceedings.

[27] Krachunov S., Beach C., Casson A.J., Pope J., Fafoutis X., Piechocki R.J., Craddock I. Energy efficient heart rate sensing using a painted electrode ECG wearable. Proceedings of the GIoTS 2017—Global Internet of Things Summit, Proceedings.

[28] Pereira C.D.M., Mendes P.M. Development of a two-electrode ECG acquisition system with dynamic interference rejection. Proceedings of the 1st Portuguese Meeting in Biomedical Engineering, ENBENG 2011.

[29] Teeramongkonrasmee A., Somboon P., Lek-Uthai A. Performance of a QRS detector on self-collected database using a handheld two-electrode ECG. Proceedings of the BMEiCON 2017—10th Biomedical Engineering International Conference.

[30] Le T., Han H.-D., Hoang T.-H. A Low Cost Mobile ECG Monitoring Device Using Two Active Dry Electrodes. Proceedings of the IEEE Sixth International Conference on Communications & Electronics.

[31] Babusiak B., Gala M., Barabas J. Design of one-lead ECG data logger. Proceedings of the 2015 38th International Conference on Telecommunications and Signal Processing, TSP 2015.

[32] Antayhua R.R., Da Silva G.M., De Sousa F.R. A duty-cycle controlled variable-gain instrumentation amplifier applied for two-electrode ECG measurement. Proceedings of the 2012 IEEE I2MTC—International Instrumentation and Measurement Technology Conference.

[33] Texas Instruments (2011). Low-Power, 2-Channel, 16-Bit Analog Front-End for Biopotential Measurements Datasheet.

[34] Haberman M., Spinelli E. A digital Driven Right Leg Circuit. Proceedings of the 2010 Annual International Conference of the IEEE Engineering in Medicine and Biology, EMBC’10.

[35] Babusiak B., Borik S., Balogova L. (2018). Textile electrodes in capacitive signal sensing applications. Meas. J. Int. Meas. Confed..

[36] Dobrev D., Daskalov I. (2002). Two-electrode biopotential amplifier with current-driven inputs. Med. Biol. Eng. Comput..

[37] Gambarotta N., Aletti F., Baselli G., Ferrario M. (2016). A review of methods for the signal quality assessment to improve reliability of heart rate and blood pressures derived parameters. Med. Biol. Eng. Comput..

[38] Zaunseder S., Huhle R., Malberg H. CinC challenge—Assessing the usability of ECG by ensemble decision trees. Proceedings of the Computing in Cardiology.

[39] Babušiak B., Borik S. (2016). Bluetooth Communication for Battery Powered Medical Devices. J. Electr. Eng..

